# Microchimerism: the hidden cellular dialogue between mother and foetal that shapes lifelong immunity

**DOI:** 10.1017/erm.2026.10061

**Published:** 2026-07-02

**Authors:** Mohammadreza Dashti, Mahtab Motevasselian, Aliyeh Niazi Moghani, Hoda Zabihi, Sajad Dehnavi

**Affiliations:** 1 Asadabad School of Medical Sciences, Asadabad, Iran; 2 Student Research Committee, Asadabad School of Medical Sciences, Asadabad, Iran; 3 Department of Obstetrics and Gynecology, School of Medicine, Shahid Akbar-Abadi and firoozgar Hospital, Iran University of Medical Sciences, Tehran, Iran; 4 Firoozgar Clinical Research and Development Center (FCRDC), Department of Obstetrics and Gynecology, School of Medicine, Firoozgar Hospital, Iran University of Medical Sciences, Tehran, Iran; 5 Kashmar School of Medical Sciences, Mashhad University of Medical Sciences, Mashhad, Iran; 6 Department of Obstetrics and Gynecology, Faculty of Medicine, Mashhad University of Medical Sciences, Mashhad, Iran; 7 Immunology Research Center, Mashhad University of Medical Sciences, Mashhad, Iran; 8 Allergy Research Center, Mashhad University of Medical Sciences, Mashhad, Iran

**Keywords:** autoimmune disease, cancer, foetal microchimerism, immune system, immune tolerance, maternal microchimerism, tissue repair

## Abstract

Foetomaternal microchimerism establishes a lifelong reciprocal exchange of immune cells that shapes immunity in both mother and offspring. Foetal microchimeric cells (FMc) include CD34^+^ progenitors, CD8^+^ CTLs and Th2-polarized lymphocytes that integrate into maternal bone marrow, spleen and inflamed tissues, promoting peripheral tolerance via Treg (FOXP3^+^CD4^+^) expansion, CTLA-4 upregulation and IL-10/TGF-β secretion, while inhibiting CD8^+^ cytotoxicity through clonal deletion and CXCR3 downregulation. Conversely, maternal microchimerism (MMc) transfers memory T cells, CD34^+^ HSCs and innate effectors (macrophages, NK cells) to the foetal thymus and lymphoid tissues, driving NIMA-specific Treg differentiation via CNS1-dependent Foxp3 induction and PD-L1^+^ DC suppression of Th1/Th17 responses. HLA class II incompatibility, complement (C3/C5a) and CCL2/CCR2 signalling coordinate this cellular crosstalk. Clinically, FMc enhances maternal tissue repair and cancer surveillance but increases autoimmune risk in scleroderma and PBC; MMc boosts newborn pathogen resistance and reduces GVHD in NIMA-matched HSCT. This review establishes microchimerism as a crucial regulator of intergenerational immunological homeostasis.

## Graphical abstract

**Figure:**
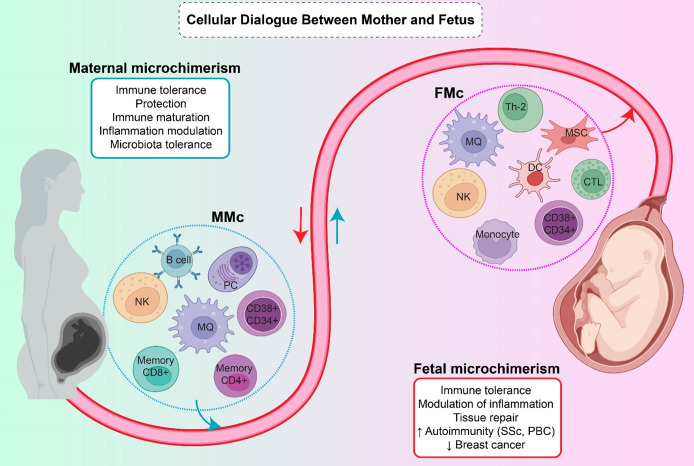

## Introduction

In ancient texts, chimera means an animal with the head of a lion, the body of a goat and the tail of a snake (Ref. 1). Today, in biology, chimera means the presence of two or more genetically different cells (originating from separate zygotes) in the body of an organism (Ref. 2). The presence of two genetically different cells in a specific organ is also called microchimerism (Ref. 2). Although different causes such as blood transfusion, solid organ transplantation, bone marrow transplantation can cause microchimerism, pregnancy, abortion and cell transfer through sexual intercourse (as a possible hypothesis without definitive evidence) can also cause it to happen, the first factors being known as artificial microchimerism and the second factors as natural microchimerism (Ref. 3).

During pregnancy, substances needed for foetal development such as nutrients, electrolytes, hormones and antibodies are transferred from the mother to the foetus, and the newborn’s waste products such as carbon dioxide and waste products from the foetus enter the mother’s body (Ref. 4). In 1893, the presence of foetal cells in the bodies of dead pregnant mothers was first observed, and further research subsequently led to the identification of foetal DNA in the bodies of pregnant mothers (Ref. 5). The use of PCR to identify the gene encoding the sex-determining region Y protein (SRY) or the DYS14 multicopy gene has been used by researchers for many years as one of the conventional methods for the Y chromosome in pregnant mothers with male foetuses (Ref. 6). Also, other research showed that foetal leucocytes can be transferred to the maternal circulation, such that in the late first trimester of pregnancy, approximately 1–6 nucleated foetal cells per millilitre of maternal blood were found. It has been observed that foetal cells are detected in the maternal bloodstream from the seventh week of pregnancy and their number increases progressively until delivery, reaching a peak of approximately 1–100 cells per millilitre of maternal blood (Ref. 7). These cells, which contain paternal antigens, persist in various maternal tissues not only during pregnancy but also for decades after delivery (reportedly up to more than 30 years) (Ref. 2). In the opposite direction, maternal cells are also transferred to the foetus from the second trimester of pregnancy (from week 13) and are localized in foetal tissues. These cells, which contain non-inherited maternal antigens (NIMAs), persist in the child after birth and play a role in the establishment and maintenance of immune tolerance to maternal antigens (Ref. 8). On the other hand, Peter Medawar, seeking a better understanding of immune tolerance, proposed his famous hypothesis of maternal tolerance to a semi-allogenetic foetus (Ref. 9). He explained why the maternal immune system, which should naturally reject any foreign tissue (such as an organ transplant), does not reject the foetus, which is genetically semi-allogeneic (due to paternal genes). Today, we know that several factors such as the blood-placental barrier, the presence of Treg cells, the lack of HLA class I and II expression on trophoblast cells, the expression of immune inhibitory molecules (such as IDO, TGF-β and IL-10) in the uterine environment, and the immunomodulatory phenotype in the uterus contribute to foetal survival (Ref. 10). However, the presence of a pathogen during pregnancy can activate cytotoxic immune cells, which indicates that the maternal immune system is performing its function properly (Ref. 11). Therefore, the presence of foetal cells in the mother’s body, known as foetal microchimerism, that may trigger various mechanisms that could have consequences for the mother’s body (Ref. 12). Although there is no strong evidence that the presence of these cells in the mother’s body can be beneficial or harmful to maternal health, research has shown that the presence of these cells in the mother’s body can play a role in tissue repair, autoimmune diseases, cancers, organ transplantation, secondary miscarriage and pregnancy complications such as pre-eclampsia (Refs 13, 14). For example, recent studies show that foetal microchimeric cells (FMc) can affect maternal lung health after delivery, and their higher levels are associated with improved lung function (Ref. 15). Also, in the field of neurobiology, these cells have been observed in association with structural changes in the maternal brain, though causality remains to be established and may play a role in mother–child neurobiological metamorphosis (Ref. 16). On the other hand, just as foetal cells can be transferred to the mother’s body, maternal cells can also be transferred to the foetus and participate in foetal health tasks such as developing and strengthening the foetal (neonatal) immune system and neonatal autoimmune diseases, which is known as maternal microchimerism (MMc) (Ref. 17). However, it is critical to note that nearly all human studies to date are correlational and demonstrating association, not causation. Whether FMc actively drives these pathologies or merely accumulates secondarily to inflammation-induced trafficking remains unresolved, a distinction that requires prospective longitudinal studies with tissue-specific sampling. Also, previous reviews have primarily addressed acute maternal–foetal cellular interactions at the placental interface, the present narrative review uniquely conceptualizes foetomaternal microchimerism as a lifelong, bidirectional cellular dialogue that persists for decades postpartum and actively programmes systemic immunity in both mother and offspring across generations. This article integrates molecular, cellular and clinical evidence to position microchimerism as an evolutionary mechanism of intergenerational immune homeostasis rather than a transient phenomenon. Although different studies have examined the different effects of FMc and MMc, in this study, we try to compile the studies conducted to gain a better and more recent understanding of foetal microchimerism and MMc, and to examine the impact of each on foetal and maternal health.

## Microchimerism and the immune system

**Figure 1.**
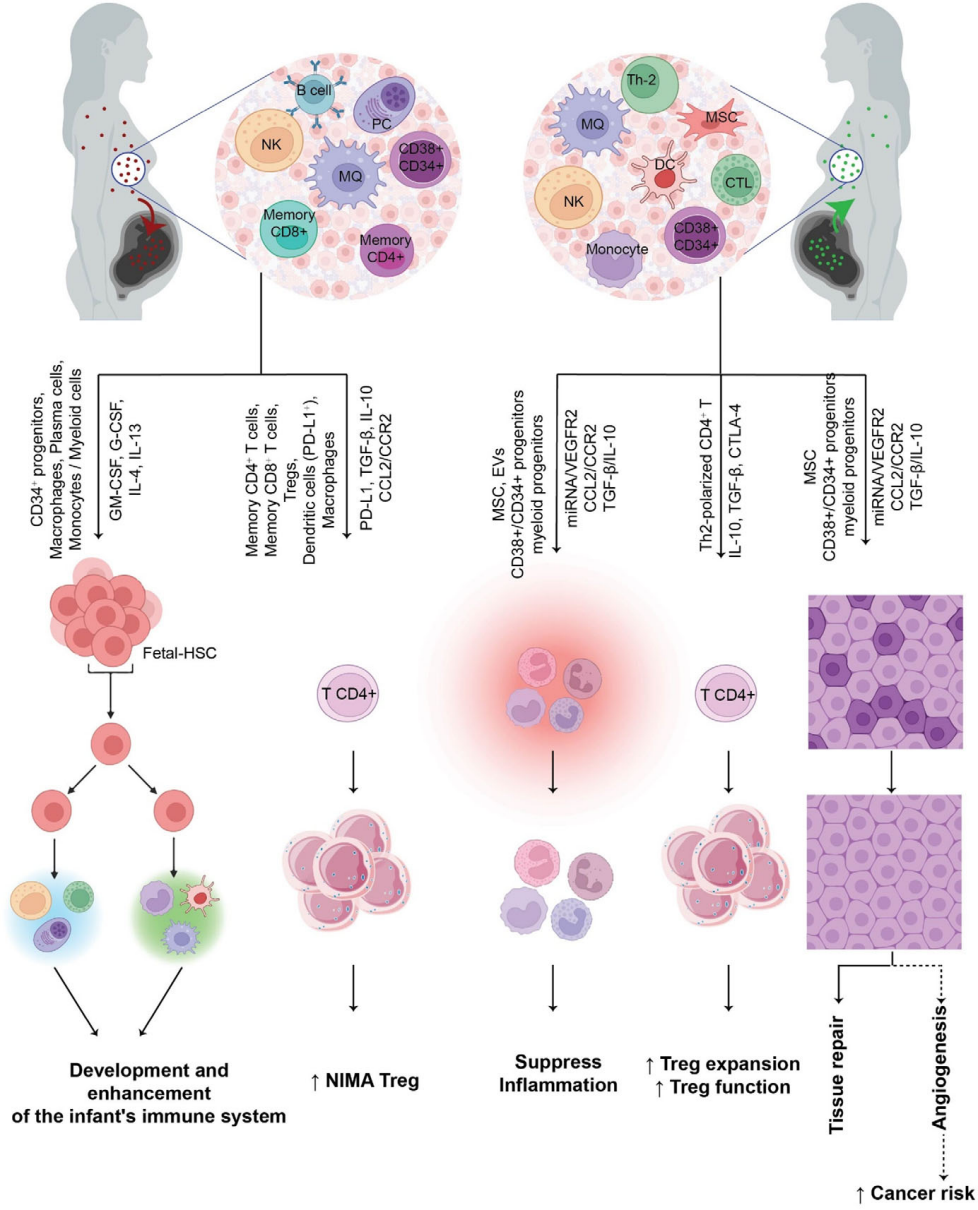
Over 20 distinct immune and progenitor populations traffic bidirectionally from gestational week 7 (FMc) and week 13 (MMc) via placenta, bloodstream and breast milk, persisting for decades. FMc primarily drives maternal tissue repair and peripheral tolerance while elevating autoimmune risk; MMc establishes lifelong NIMA-specific tolerance, accelerates neonatal immune maturation and confers clinical benefit in NIMA-matched HSCT through reduced GVHD and enhanced GvL. Key mediators include TGF-β, IL-10, PD-L1, CCL2/CCR2, HLA mismatch and EV-transported miRNA. Figure 1. long description.

After crossing the placenta as early as gestational week 7, FMc do not simply persist as passive remnants and they actively remodel maternal immunity. As outlined in Figure 1, FMc are predominantly CD34^+^/CD38^+^ haematopoietic progenitors that present non-inherited paternal antigens (IPA) to the maternal immune system, functioning as endogenous antigen-presenting cells (Ref. 37). Mechanistically, CCR2^+^ foetal cells home to inflammatory sites, where they instruct maternal macrophages to secrete IL-10, accelerating wound healing (Ref. 38). Simultaneously, FMc accumulating in maternal bone marrow and spleen drive clonal deletion of CD8^+^ cytotoxic T lymphocytes and downregulate CXCR3 expression; two complementary mechanisms that enforce central and peripheral tolerance through HLA class II mismatches (Ref. 39). In systemic sclerosis (SSc), women harbour elevated FMc levels compared to healthy controls. This increase correlates with upregulation of TGF-β-driven fibrotic pathways and diminished NK cell activity, implying that FMc may act as chronic immune activators rather than passive bystanders (Ref. 40). During murine pregnancy, rising FMc levels in maternal blood parallel the expansion of CD4^+^FOXP3^+^ Tregs. These Tregs, generated via peripheral induction in a CNS1-dependent manner, establish tolerance to paternal antigens and protect against foetal rejection (Ref. 41). FMc in maternal tissues further enforce peripheral tolerance by suppressing dendritic cell function and upregulating CTLA-4 on T lymphocytes, a mechanism that simultaneously supports tissue repair and limits fibrosis (Ref. 42). In primary biliary cholangitis (PBC), FMc is detectable in damaged livers at levels comparable to other liver diseases. However, higher FMc burden correlates with HLA class II compatibility between mother and offspring, which suppresses CD4^+^ T cell activity and skews the cytokine balance towards Th2 (IL-4/IL-13) (Ref. 43). In Hashimoto’s thyroiditis, male foetal DNA is more abundant in diseased thyroid glands compared to healthy controls, paralleling reduced IFN-γ and elevated TGF-β. This pattern positions FMc as a potential chronic driver of autoimmune pathology (Ref. 18). Collectively, FMc are not mere pregnancy memories; they actively maintain lifelong immune homeostasis. This insight has clinical traction in HSCT, where HLA class II mismatch between chimeric and host cells correlates with reduced rheumatoid arthritis severity during pregnancy and informs donor selection (Ref. 21). MMc, the transfer of maternal cells to the foetus starting in the early second trimester, primarily shapes foetal immune development (Ref. 18). These cells, predominantly memory T lymphocytes and CD34^+^ HSCs, home to the foetal spleen and thymus, where they promote tolerance to paternal antigens via non-inherited maternal antigen (NIMA) expression (Ref. 18). MMc in cord blood correlates with expanded Treg populations and attenuated Th1/Th17 responses, driven by direct suppression of IFN-γ and IL-17 production (Ref. 44). In HSCT models, NIMA matching lowers acute graft-versus-host disease (aGvHD) risk, an effect attributed to crosstalk between donor cells and recipient NK cells/monocytes, alongside upregulated PD-L1 expression (Ref. 45). In pre-eclampsia, reduced MMc levels parallel heightened systemic inflammation and Th1/Th17 hyperactivity, underscoring a protective role for MMc in immune homeostasis (Ref. 46). MMc are enriched in memory T and NK cell subsets. Through CCL2/CCR2 signalling, they home to inflamed tissues and drive naïve T-cell differentiation into Tregs, a property that reduces graft rejection in HSCT (Ref. 47). In cord blood, MMc associate with increased naïve and memory CD4^+^ T cells while suppressing Th1 responses via IFN-γ inhibition. In animal models, MHC class II-mismatched maternal cells further promote CD4^+^ T cell differentiation into Tregs through peripheral tolerance pathways (Ref. 48). Reduced MMc in pre-eclampsia also correlates with elevated IL-17/Th17 activity and diminished dendritic cell PD-L1 expression, reinforcing its protective role (Ref. 49). In HSCT, NIMA matching accelerates recovery of CD4^+^CD25^+^CD45RA^+^ Tregs by day 30 post-transplant and reduces grade II–IV acute GvHD. Moreover, KIR ligand mismatch between donor and recipient NK cells enhances graft-versus-leukaemia (GvL) effects without elevating GvHD risk (Ref. 50). Table 1 summarizes the key cellular and molecular mediators of this complex dialogue. Collectively, these findings highlight the context-specific nature of microchimerism: the same cells can drive repair or pathology depending on the microenvironment, a duality that opens therapeutic avenues in autoimmunity and transplantation.

**Table 1.** Molecular mediators and cytokine balance shaping foetomaternal immune tolerance Table 1. long description.

Molecular pathway	Key cytokines/molecules	Primary cellular source	Biological source (maternal/foetal origin)	Functional effect	Immunological interpretation	References
TGF-β/Smad axis	TGF-β, Smad2/3, Foxp3	Tregs, decidual macrophages	Predominantly maternal (decidua, uterine immune cells)	Induction of regulatory T cells and inhibition of effector Th1/Th17 activity	Central pathway maintaining pregnancy tolerance and fibrosis control	(Refs 18–20)
IL–10/CTLA–4 pathway	IL–10, CTLA–4, IDO	Maternal DCs, FOXP3^+^ T cells	Maternal (decidua and peripheral circulation)	Suppresses antigen presentation and dampens cytotoxic activity	Prevents foetal rejection through peripheral immune quiescence	(Refs 21–23)
Th1/Th2/Th17 polarization	IFN-γ, IL–4, IL–13, IL–17	CD4^+^ T helper subsets	Both maternal and foetal (shared cytokine milieu)	Th2 dominance promotes tolerance; Th1/Th17 activation drives pathology	Dynamic equilibrium determines pregnancy success or autoimmunity	(Refs 24–26)
Chemokine signalling	CCL2/CCR2, CXCL9/10, CXCR3	Foetal myeloid progenitors, maternal leucocytes	Bidirectional (foetal cells secrete CCL2; maternal cells express CCR2)	Guides cell trafficking to inflamed or reparative sites	Directs the spatial dialogue between tolerance and inflammation	(Refs 27, 28)
PD-L1/PD–1 checkpoint	PD-L1, PD–1, CD80/86	DCs, trophoblasts, T cells	Both dominant foetal trophoblast expression	Inhibits T-cell activation and fosters immune exhaustion	Recreates ‘immune silence’ at the maternal–foetal interface	(Refs 29–31)
HLA-G/Complement system	HLA-G, C3, C5a	Trophoblasts, macrophages	Primarily foetal (trophoblastic origin)	Blocks complement-mediated lysis and promotes NK cell tolerance	Establishes immune privilege and protection against graft-like attack	(Refs 32, 33)
EV-mediated miRNA transfer	miR–146a, miR–155, TGF-β cargo	MSCs, placental EVs	Predominantly foetal, but also maternal feedback EVs	Epigenetic suppression of NF-κB and inflammatory genes	A post-transcriptional mechanism of intercellular immune education	(Refs 34–36)

This table summarizes the molecular pathways, cytokines, cellular sources and major functions that contribute to the establishment and maintenance of a tolerogenic immune environment during pregnancy. The pathways range from T-cell tolerance (e.g., the TGF-β/Smad and IL-10/CTLA-4 axis) and cytokine balance (Th1/Th2/Th17 polarization) to inhibitory point interactions (e.g., PD-L1/PD-1), chemokine signalling to direct cellular traffic and unique mechanisms such as HLA-G expression by the placenta and miRNA transfer via extracellular vehicles (EVs). This complex network ensures a regulated immune environment that both protects the foetus from maternal immune attacks and mediates anti-inflammatory responses.

Before dissecting the role of individual immune lineages, it is essential to define what constitutes a ‘cellular dialogue’ at the molecular level. In the context of foetomaternal microchimerism, dialogue refers to reciprocal, ligand-receptor-mediated signalling events between transferred chimeric cells (FMc or MMc) and host tissues that result in functional reprogramming, not merely physical co-existence. As summarized in Table 2, this dialogue operates through four major molecular platforms: (1) cytokine-receptor signalling (e.g., TGF-β/TGF-βR → Smad2/3 → FOXP3 induction); (2) immune checkpoint interactions (e.g., PD-L1/PD-1 → SHP-2 activation → T-cell anergy; CTLA-4/CD80/86 → IDO upregulation); (3) chemokine-guided homing (e.g., CCL2/CCR2 → PI3K/ERK → recruitment to inflamed tissues) and (4) extracellular vesicle (EV)-mediated transfer of regulatory miRNAs (e.g., miR-146a → TLR7/NF-κB suppression). Unlike classical transplantation, where allorecognition leads to rejection, the foetomaternal dialogue is uniquely biased towards tolerance induction through the concurrent engagement of multiple inhibitory pathways. The following subsections (foetomaternal microchimerism and Tregs; foetomaternal microchimerism and CTL, Th1 and Th2; foetomaternal microchimerism and B lymphocytes; foetomaternal microchimerism and innate immune cells; foetomaternal microchimerism and HLA alleles and foetomaternal microchimerism and MSC and their EVs) re-examine each immune lineage through this mechanistic lens.

**Table 2.** Temporal dynamics of microchimerism: from seeding to systemic imprinting Table 2. long description.

Trimester	FMc frequency/burden	Dominant phenotypes	Key molecular drivers	Immunological outcome	References
First	Detectable (4–6 weeks)	CD34^+^, CD38^+^ (stem-like)	TGF-β/Smad, SOX2, NANOG	Central/peripheral tolerance; NIMA-specific Tregs	(Refs 4, 51, 52)
Second	~1 per 1,000 PBMCs	VEGFR2^+^, CD31^+^ (mesenchymal)	Th2-cytokines, miRNA-EVs	Thymic education; diversification; pregnancy support	(Refs 4, 45)
Third	Peak (>100 cells/mL)	Mature Effectors (NK, macrophages)	miRNA-loaded EVs, CffDNA	Systemic imprinting; postpartum repair; transgenerational benefits	(Refs 51, 53, 54)

### Foetomaternal microchimerism and Tregs

#### FMc and Treg

The cellular dialogue of microchimerism is orchestrated by multiple immune lineages acting in concert. We begin with regulatory T cells (Tregs), which serve as the primary architects of tolerance, before examining how cytotoxic T lymphocytes (CTLs) provide immune surveillance and how B cells, innate cells and molecular mediators (HLA, complement, EVs) collectively shape the functional outcome. In reproductive immunology, CD4^+^ Tregs are central to tolerating the semi-allogeneic foetus. Defined by high FOXP3 expression, they suppress effector T-cell-driven inflammation (Ref. 25). In mice, selective depletion of MMc reduces NIMA-specific Tregs and increases foetal loss. Conversely, MMc presence in female offspring enhances survival of third-generation descendants genetically similar to the grandmother, a transgenerational fitness benefit (Ref. 55). Maternal tolerance to the foetus requires rapid immune adaptation: suppressive CD4^+^ Tregs expand immediately after conception, paralleling increasing foetal antigen load (Refs 19, 20, 25).

In animal models, foetal loss correlates with either insufficient Treg accumulation (in abortion-prone inbred matings) or reduced per-cell Treg suppressive capacity during prenatal infection (Ref. 23). A major knowledge gap persists: most murine studies measure total Tregs, not NIMA-specific Tregs. Since only a fraction of Tregs are foetal-specific, reported reductions likely overestimate the true contribution of foetomaternal tolerance. Future work must use tetramer-based sorting of NIMA-specific Tregs to establish causality. Allogeneic pregnancies expand maternal Tregs more robustly than syngeneic pregnancies, underscoring the role of maternal–foetal MHC disparity in Treg accumulation (Refs 20, 25). The blunted Treg response in syngeneic settings may reflect maternal mismatch to Y-encoded alloantigens or self-antigen stimulation, aligning with increased demands for tissue repair during pregnancy (Refs 27–29). In mice, restoring maternal Tregs to pre-pregnancy levels causes foetal loss only in allogeneic, not syngeneic, pregnancies, proving that elevated Treg counts are essential for allogeneic foetal survival (Refs 20, 30). In human pregnancy, restricted foetal growth and altered Treg accumulation associate with pre-eclampsia and spontaneous abortion but causation versus correlation remains unresolved (Ref. 31). Conversely, foetal antigen-specific maternal CD4^+^ T cells proliferate and preferentially differentiate into Tregs, marked by FOXP3 upregulation (Ref. 30). Selective blockade of Treg differentiation in foetus-specific maternal CD4^+^ T cells trigger foetal loss, proving these cells are essential for murine pregnancy success (Ref. 32). FMc secrete TGF-β and IL-10, which bind maternal TGF-βRII and IL-10R to trigger Smad2/3 phosphorylation and CNS1-dependent Foxp3 induction in CD4^+^ T cells, while upregulating CTLA-4 to inhibit APC costimulation.

From gestational week 10, human foetal T cells populate lymphoid tissues and acquire alloantigen-induced proliferation capacity (Refs 33, 34). Yet they do not attack maternal tissues. Instead, NIMA stimulation drives foetal CD4^+^ T cells towards Treg differentiation, and this expanded Treg pool suppresses NIMA-specific effector T cells, preventing maternal–foetal conflict (Ref. 34). In mice, MMc depletion rapidly reduces NIMA-specific FOXP3^+^CD4^+^ Tregs, indicating that continuous postnatal NIMA exposure sustains these Tregs in offspring (Ref. 55). Foetal protection correlates with expansion of NIMA-specific maternal Tregs (which partially overlap with paternal–foetal specificity), not with foetus-only Tregs. MMc removal abolishes this protective effect in subsequent pregnancies and eliminates NIMA-specific Treg accumulation (Ref. 55). NIMA-specific tolerance confers intergenerational reproductive benefits. Beyond Mendelian inheritance of maternal genes, vertical transmission and postnatal persistence of maternal cells reinforce tolerance to non-inherited maternal traits in subsequent pregnancies (Ref. 55).

Persistent FMc in parous women may similarly enhance tolerance in subsequent pregnancies (Ref. 35). Human data support this: pre-eclampsia risk drops in second pregnancies with the same (but not a new) partner. This partner-specific benefit correlates with persistent foetus-specific maternal Tregs after delivery, their rapid recall expansion upon re-exposure to foetal antigens, and enhanced protection against Treg-depletion-induced foetal loss in secondary versus primary pregnancies (Ref. 30). Thus, persistent FMc may represent a selfless evolutionary gesture: firstborns promote maternal acceptance of genetically similar future siblings at the expense of tension with dissimilar ones.

Pregnancy-driven Treg expansion may link reproductive hormones to protection against autoimmune relapse. In murine multiple sclerosis models, glucocorticoid receptor signalling in maternal T cells induces peripheral Tregs and prevents paralysis (Ref. 36). These connections call for further research into how FMc influences maternal Treg expansion during pregnancy. In postpartum women, foetus-specific CD8^+^ T cells with regulatory function have been isolated alongside conventional foetus-specific CTLs. Using a murine collagen-induced arthritis (CIA) model, Chang et al. showed that pregnancy ameliorates arthritis progression (Ref. 56). FMc were detected in maternal bone marrow and blood, but protection was not Treg- or Tc-mediated. Instead, it correlated with the expansion of CD14^+^IL-10^+^ cells in bone marrow. Thus, FMc confer protection against inflammatory arthritis by inducing immunosuppressive CD14^+^IL-10^+^ cells, a novel immunomodulatory mechanism (Ref. 56). In a paternal intrauterine transplantation (IUT) model, successful engraftment correlated with elevated MMc (CD4^+^, CD8^+^ and CD19^+^) and donor-specific tolerance (Ref. 57). Humoral responses remained low (IgM/IgG), but Tregs expanded, while pro-inflammatory cytokines were suppressed upon paternal antigen exposure. Thus, paternal IUT creates a Treg-dominated, muted humoral environment that enhances engraftment, illustrating how foetal material simultaneously triggers tolerance and immunity, with the net outcome dictated by their equilibrium (Ref. 58).

#### MMc and Treg

Newborns face high risks from microbial infection. MMc may enhance foetal immune maturation by supplying growth and differentiation factors. Alternatively, expanded NIMA-specific Tregs could improve offspring health by dampening perinatal inflammation and suppressing aberrant immune responses that drive allergy and autoimmunity (Refs 59–61). Also, NIMA-specific Treg induction by maternal microchimeric cells not only complements the peripheral tolerance established by foetal cells in the mother but also sets the stage for innate immune cells to fine-tune this tolerance at mucosal and inflammatory sites. Pathogens like the hepatitis B virus exploit this developmental tolerance window, establishing persistent infection more effectively after vertical transmission (Ref. 62). In mice, the risk of foetal loss from tolerance disruption (e.g., *Listeria* infection or Treg depletion) is significantly lower in pregnancies fathered by NIMA-matched versus NIMA-mismatched males (Ref. 55).

In both humans and mice, pregnancy and breastfeeding expose offspring to MMc and HLA molecules, inducing long-lasting T-cell-mediated tolerance to NIMA (Ref. 63). MMc in foetal lymph nodes promote NIMA tolerance by driving development of foetal CD4^+^CD25ʰⁱᵍʰFOXP3^+^ Tregs, which calibrate foetal immune responses to maternal cell invasion (Ref. 23). NIMA tolerance has major clinical implications: in solid organ transplantation, NIMA-mismatched donors confer superior tolerance over IPA; child-to-mother bone marrow transplants reduce GVHD-related morbidity/mortality and renal grafts from NIMA-mismatched siblings survive longer than non-mismatched grafts. In mice, targeted deletion of CNS1 (essential for Foxp3 induction) causes foetal loss, underscoring its mechanistic importance (Ref. 64). Human colostrum contains ~80% myeloid leucocytes (40%–50% macrophages, 5%–10% neutrophils) and ~20% lymphoid cells, of which T cells predominate (>80% of lymphoid fraction), with minor B, NK and innate lymphoid cell populations (Ref. 65). In human milk, γδ TCR^+^CD8^+^ T cells outnumber γδ CD4^+^ and αβ CD4^+^/CD8^+^ T cells (Ref. 66). Milk T cells express mucosal homing markers (l-selectin/CD62L, integrin α4β7, MAdCAM-1, integrin αEβ7/CD103 and CD223/LAG-3), indicating migration from maternal mucosa to mammary gland (Refs 67, 68). Integrin α5β7, another mucosal marker, is more abundant on milk CD8^+^ T cells than on their blood counterparts (Ref. 69).

A subset of milk T cells is CD127^−^CD25^++^FOXP3^+^ Tregs that express high TGF-β (Ref. 70). These maternal Tregs independently shape offspring immunity, fostering tolerance to maternal cells and gut microbiota by inducing anergy or tolerance in developing T lymphocytes (Ref. 71). Human milk contains high TGF-β levels, which stabilize FOXP3 expression and sustain Treg differentiation, thereby enhancing neonatal mucosal tolerance through improved intestinal integrity and microbial colonization (Refs 72, 73). Wood et al. showed that at 3 weeks of age, exclusively breastfed infants have twice the Treg fraction of formula-fed neonates (Ref. 63).

Upon antigen exposure, Tregs proliferate faster than CD8^+^ T cells recognizing the same antigen, thereby limiting CD8^+^ T cell expansion (Ref. 74). Thus, NIMA-specific maternal Tregs in milk limit expansion of NIMA-specific CD8^+^ T cells and promote MMc persistence into adulthood. In murine models, pregnancy exposure to male antigens induces tolerance to syngeneic male skin grafts and abrogates cytotoxic responses against these antigens (Ref. 75). The key mediator is pregnancy-induced Tregs, which prevent foetal allograft rejection and foster tolerance to foetal antigens (Ref. 76).

Foetomaternal microchimerism has transformed HSCT for haematological disorders (Refs 45, 77). Donor compatibility and GVHD risk remain barriers, but microchimerism-induced tolerance enables haploidentical transplantation using family donors. NIMA-mismatched donors confer significantly lower severe rejection rates than IPA-mismatched donors (Ref. 78). Postnatal persistence of Tregs that suppress responses to in utero NIMA is critical. Maintaining MMc into adulthood requires ongoing oral NIMA exposure via breastfeeding from antigen-positive mothers, which sustains higher NIMA-specific Tregs than effector T cells (Ref. 79). Maternal alloantigens drive Treg proliferation in foetal lymph nodes via TGF-β (Ref. 26). Newborn mice nursed by surrogate mothers unexposed to NIMA lose tolerance to NIMA-expressing cells and eventually deplete microchimerism (Ref. 21). Ingested maternal MHC antigens in milk generate additional NIMA-specific Tregs that inhibit NIMA-specific effector T-cell proliferation and sustain microchimerism (Ref. 21). MMc correlate with NIMA-specific Tregs that suppress delayed-type hypersensitivity and lymphoproliferation of NIMA-specific effector T cells in adult mice (Ref. 80). Some Tregs differentiate into long-lived memory T cells, enabling sustained suppression of NIMA antigen responses (Ref. 67). Immune cells cross the placenta into human foetal lymph nodes, where they promote Treg development and suppress responses to maternal antigens (Ref. 64). Beyond NIMA tolerance, this process may foster tolerance to other foreign and self-antigens encountered in utero, shaping offspring immune development. Collectively, these mechanisms, preferential Treg differentiation (TGF-β/CNS1), NIMA-specific Treg persistence and breastfeeding-mediated tolerance maintenance, form an intricate network that stabilizes foetomaternal microchimerism as an evolutionary strategy for genetic perpetuation and intergenerational immune regulation, while laying the groundwork for future therapeutic and transplant applications.

### Foetomaternal microchimerism and CTL, Th1 and Th2

If Tregs are the brakes of the microchimeric dialogue, CTLs, Th1 and Th2 are the accelerator. CD4^+^ T cells polarize into Th1 (IFN-γ/IL-2, cellular immunity) or Th2 (IL-4/IL-5/IL-13, humoral immunity). CD8^+^ CTLs, armed with granzyme and perforin, provide cytotoxicity against infected or malignant cells – but can also challenge foetal tolerance (Refs 48, 68). In mice, maternal exposure to male foetal antigens during pregnancy induces CTL tolerance to syngeneic male skin grafts, an effect mediated by Treg induction during antigen exposure (Ref. 70). Using HLA tetramers containing SMCY epitopes, HY-specific maternal CTLs are detectable in 37% of parous women, rising to 50% in mothers with two or more sons. These functional CTLs produce IFN-γ and lyse HLA-compatible male (but not female) cells (Ref. 81). Recently, postpartum women were found to harbour not only foetus-specific cytotoxic CTLs but also foetus-specific CD8^+^ T cells with regulatory functions, revealing a tunable balance between tolerance and immunity that shapes maternal health outcomes (Ref. 82).

Maternal immune responses to FMc follow transplantation biology principles: direct (interaction with foetal APCs) or indirect (presentation of foetal peptides by maternal cells) (Ref. 83). Even rare foetal CTLs in maternal circulation can directly recognize maternal antigens and reshape the immune environment via cytokine secretion (Ref. 83). Murine models further illustrate that clonal deletion within the thymus effectively removes self-reactive CTLs, thereby preserving peripheral tolerance (Ref. 84). In transplantation, parous female donors have elevated GVHD risk because pregnancy sensitizes T cells to paternal minor histocompatibility antigens (Refs 81, 85). However, this sensitization also enhances graft-versus-tumour effects (Ref. 86), and multiparous donors lower leukaemia relapse risk (Ref. 87). Therefore, understanding FMc thus enables donor selection that balances GvL and GVHD.

In secondary recurrent miscarriage following a live birth, T-cell immunity targets Y-chromosome antigens (Ref. 88). A firstborn son primes maternal CTLs against Y-antigens, which may attack a subsequent male foetus (Ref. 89). Dutch cohort studies show that a male firstborn reduces subsequent live birth rates (Ref. 90). Women with HLA class II capable of presenting Y epitopes have lower live birth rates, an effect absent after a firstborn daughter (Ref. 91). Th1-derived IFN-γ drives inflammation, tilting the balance away from Th2-mediated humoral tolerance towards miscarriage.

In SSc, foetal-origin CTLs exhibit a Th2-oriented profile (IL-4/IL-13), consistent with disease pathophysiology (Ref. 92). However, FMc in T cells is prevalent in both SSc patients and healthy controls, with study variability likely reflecting technical differences (Refs 93–95). In PBC, Y-chromosome-positive cells appear in liver biopsies of 8/19 patients, though other studies find no definitive link (Ref. 96). In Sjögren’s syndrome, foetal cells are detectable in exocrine gland lesions but not in peripheral blood (Ref. 89). Nested PCR reveals higher male DNA frequency in salivary glands (Ref. 97). In both Hashimoto’s and Graves’ thyroiditis, FMc is increased in the thyroid gland but not in peripheral blood (Ref. 98). In SLE, peripheral blood PCR shows elevated microchimerism (Ref. 99), though other studies find no correlation with disease activity (Ref. 100). HLA class II compatibility between SSc patients and their offspring enhances FMc, likely by dampening alloreactive responses (Ref. 101). In JIIM, MMc is elevated in blood and muscle lesions (Refs 92, 102). In neonatal lupus, alpha-actin^+^ maternal cells are detectable in heart block lesions (Ref. 103).

In cancer, maternal CTLs targeting minor histocompatibility antigens (e.g., HA-1) enhance immune surveillance and reduce breast cancer risk. Parous women have lower breast cancer incidence and better survival/treatment responses (Ref. 104). In breast cancer patients, FMc is diminished (Ref. 105). An alternative explanation, rarely considered, is that reduced FMc reflects successful immune clearance of foetal cells by an activated maternal immune system, not failed trafficking. Distinguishing reduced transfer from enhanced clearance requires kinetic studies with labelled foetal cells (feasible in non-human primates). In pregnancy-associated high-grade breast carcinomas, foetal cells expressing HLA-G accumulate in the stroma, promoting immune evasion and impacting pathogenesis (Ref. 106). A murine model corroborates increased foetal cell numbers in high-grade tumours (Ref. 107). Foetal progenitor cells home to melanomas, contributing to blood vessel and lymphatic formation and promoting tumour angiogenesis (Refs 108, 109). In chemically induced skin tumours, 75% are FMc-positive with stroma development (Ref. 110). In pregnancy-associated melanomas, 63% show FMc and enhanced VEGF-driven angiogenesis (Ref. 111). Protective FMc effects are reported in breast, bladder, ovarian, brain, pancreatic and colorectal cancers, as well as lymphoma and leukaemia (Ref. 112). However, in the thyroid, skin and breast, a repair mechanism is proposed instead of a role related to cancer stem cells (Refs 113, 114).

In tissue repair, foetal CTLs migrate to injury sites. Post-delivery, they appear in the spleen, liver and kidney after hepatic/renal injury (Refs 115, 116) and can cross the blood–brain barrier following brain injury (Ref. 117). In pregnancy-associated appendicitis, male cells are more prevalent at inflammation sites (Ref. 110). Recovery from late-pregnancy myocardial infarction is remarkably enhanced. In murine models, foetal cells migrate to the injured heart and differentiate into cardiomyocyte-like, endothelial and smooth muscle cells expressing connexin 43 and troponin T (Refs 118, 119). Cdx2^+^ fetoplacental cells further improve contractile function (Refs 120–122). In damaged skin, FMc persist in the epidermis as keratinocytes (Ref. 123); CCL2/CCR2 signalling recruits foetal myeloid progenitors and promotes neovascularization (Ref. 38). In fibrotic wounds, FMc persist into late remodelling stages, potentially contributing to improper healing (Ref. 38). While these findings are striking, most evidence comes from immunodeficient mouse models where rejection is artificially prevented. Whether similar differentiation occurs in immunocompetent mothers, where alloreactive T cells might eliminate foetal cardiomyocytes, remains unknown. Moreover, translation to human therapy faces the challenge of sourcing sufficient foetal cells without ethical violations, making iPSC-derived ‘pseudofetal’ cells a more practical alternative.

In immune transfer to offspring, milk-derived maternal CD8^+^ T cells, comprising 75% of milk T cells, compensate for immature neonatal CTLs by producing cytotoxic mediators (Ref. 124). These activated CD8^+^ T cells express gut-homing receptors (CD103, α4β7, CCR9), priming them for mucosal migration (Refs 69, 124). In the *Nippostrongylus brasiliensis* (Nb) model, milk-derived maternal Th2-polarized CD4^+^ T cells migrate to offspring spleen, lungs and lymph nodes, activating effector B cells and sustaining immunity into adulthood (Ref. 125). Maternal CD4^+^ T cells also seed the thymus and stimulate antigen-specific CD8^+^ T cells (Ref. 126). In preterm birth, Th1 cells drive pulmonary inflammation via IFN-γ, causing airway hyperresponsiveness and elevated IL-6/TNF-α. In milk, Th2 cells facilitate tolerance to gut microbiota through the action of IL-4 (Ref. 72). In summary, CTLs, Th1 and Th2 form a network spanning foetal rejection, cancer surveillance and tissue repair: CTLs mediate direct lysis, Th1 drives cellular inflammation and Th2 promotes humoral tolerance. We next examine how innate immune cells (macrophages, DCs, NK cells, neutrophils) initiate these adaptive responses.

### Foetomaternal microchimerism and B lymphocyte

If T cells orchestrate cellular immunity, B cells and antibodies provide the humoral arm of the microchimeric dialogue. Beyond producing protective antibodies, B cells serve as APCs that shape T-cell responses, bridging innate and adaptive immunity. In foetomaternal microchimerism, B cells promote tolerance, pathogen defence and intergenerational immune memory transfer. Maternal B cells (CD19^+^), including IgG-secreting plasma cells, cross the placenta and breast milk, compensating for neonatal adaptive immaturity and establishing long-term humoral protection (Ref. 118). In B cell-deficient (μ^−^/^−^) mice, maternal IgG-secreting plasma cells persist in offspring spleen and bone marrow for up to 24 weeks, restoring serum IgG in 70% and enabling functional humoral responses (Ref. 118). These transferred B cells express class-switched memory phenotypes (IgD^−^CD27^+^), GALT homing markers (CD44, α4β7, α4β1) and spontaneously secrete IgG, enabling antigen-specific immunity independent of offspring B-cell development (Ref. 127). In immunodeficient foetuses, maternal B cells engraft in the thymus and spleen, potentially differentiating from c-kit^+^ maternal HSCs in offspring bone marrow (Refs 128, 129).

In human milk, B lymphocytes comprise 6% ± 4% of leucocytes, predominantly activated memory cells migrating via the gut-mammary gland axis and expressing mucosal homing receptors (Refs 127, 130). These B cells present antigens to developing T cells, compensating for neonatal antigen presentation deficits and priming Th2-biased responses (IL-4/IL-13) that dampen inflammation and promote NIMA tolerance (Ref. 73). Milk-derived B cells upregulate CCR9 and α4β7, enabling transepithelial migration across the immature neonatal gut barrier (with loose tight junctions) and persisting in Peyer’s patches and extra-lymphoid organs until weaning (Refs 124, 131, 132). In murine models, milk-derived maternal B cells establish microchimerism in offspring GALT, enhancing local IgA and suppressing effector T-cell proliferation via TGF-β, preserving maternal cell engraftment into adulthood (Refs 34, 80, 133). Breastfeeding is critical: surrogate nursing eliminates NIMA-specific tolerance, causing loss of microchimerism and heightened hypersensitivity (Ref. 80).

In recurrent miscarriage and pregnancy complications, dysregulated B-cell responses drive alloantibody-mediated rejection. Elevated anti-paternal IgG antibodies correlate with placental dysfunction in pre-eclampsia and preterm labour, where increased foetomaternal trafficking amplifies humoral alloreactivity (Ref. 128). Conversely, protective antibodies from prior pregnancies (e.g., against Y-linked minor antigens) may prime humoral tolerance in subsequent gestations, reducing secondary miscarriage risk when HLA compatibility favours NIMA over IPA. In autoimmune diseases, foetal B cells are implicated in pathogenesis in some studies. In SSc, foetal-derived CD19^+^ B cells accumulate in skin lesions with a Th2-oriented profile, potentially driving autoantibody production via chronic GVHD-like mechanisms (Refs 132, 134). Study variability reflects tissue-specific distribution; foetal B cells are more prevalent in inflammatory sites (e.g., salivary glands in Sjögren’s) than in blood, highlighting HLA class II compatibility in engraftment and autoantibody diversification (Refs 135, 136). In JIIM, maternal B microchimerism in muscle lesions correlates with disease severity, possibly via pathogenic IgG accumulation (Refs 131, 132, 137). In neonatal lupus syndrome (NLS), maternal IgG autoantibodies cross the placenta, causing congenital heart block, yet transferred maternal plasma cells may alleviate severity by neutralizing pro-inflammatory cytokines (Ref. 138). This dual role (pathogenic autoantibodies vs. protective plasma cells) creates a therapeutic paradox: complete B-cell depletion (e.g., rituximab) might worsen NLS by removing protective plasma cells while sparing pathogenic autoantibodies. A nuanced strategy targeting only pathogenic clones with anti-idiotype antibodies or antigen-specific immunoadsorption remains experimental.

In cancer, B cells and antibodies provide immunosurveillance. Parous women with FMc have reduced breast cancer risk, attributed to enhanced humoral responses against tumour antigens (e.g., HA-1) where foetal B cells prime maternal IgG for ADCC (Refs 85, 105, 139). In high-grade breast tumours, foetal B-lineage cells in stroma express non-tumoral markers, suggesting a reparative (not oncogenic) role via VEGF modulation (Refs 96, 106, 107, 114). Protective effects extend to ovarian, colorectal and leukaemic cancers via complement activation and phagocytosis. In thyroid cancer, foetal B cells in follicular adenomas may drive pathogenesis via autoantibody mimicry, though evidence favours repair. In skin malignancies like melanoma, milk-derived maternal B cells transfer anti-viral IgG, reducing vertical transmission risks and indirectly protecting against tumour-promoting infections (Refs 111, 140).

In tissue repair, B cells contribute via antibody-mediated opsonization and cytokine signalling. In animal models, foetal-derived plasma cells in injured maternal liver secrete IgG that recruits macrophages, enhances debris phagocytosis and promotes resolution (Refs 115, 116). In late-pregnancy myocardial infarction, high recovery rates correlate with foetal B-lineage progenitors differentiating into cardiomyocytes, supported by maternal antibodies that stabilize neo-angiogenesis via connexin 43 (Refs 120–122). In wounded skin, CCL2-recruited foetal B progenitors transdifferentiate into endothelial cells, aiding re-epithelialization, though persistence in fibrotic wounds may perpetuate aberrant healing (Ref. 38). Milk B-cell antibodies neutralize mucosal pathogens, accelerating neonatal tissue homeostasis (Refs 141, 142).

In the Nb model, milk-derived class-switched B cells migrate to the offspring’s spleen and GALT, activating effector responses and sustaining anti-helminth IgG into adulthood, independent of pregnancy effects (Ref. 125). In μ^−^/^−^ offspring, maternal plasma cells maintain IgG against opportunistic pathogens like Epstein–Barr virus, compensating for B-cell deficiency (Refs 118, 119). Milk-derived stem/progenitor B-lineage cells (CD34^+^CD133^−^) differentiate into erythroid and lymphoid progenitors in neonatal bone marrow, expressing pluripotency genes (OCT4, NANOG) for multilineage support without tumorigenicity (Refs 136, 143). TGF-β from milk B cells induces anergy in neonatal T cells, suppressing anti-maternal responses and promoting microbiota tolerance (Refs 72, 133). In summary, B cells and antibodies in foetomaternal microchimerism orchestrate humoral networks spanning tolerance (IgG class-switching, homing receptors), cancer suppression and regenerative signalling. TGF-β and HLA compatibility evolutionarily fortify maternal–offspring health, informing antibody-based therapies in autoimmunity, transplantation and neonatal immunity.

### Foetomaternal microchimerism and Innate immune cells

Macrophages, dendritic cells (DCs), NK cells, neutrophils and monocytes form the innate first line of defence in foetomaternal microchimerism. Unlike adaptive lymphocytes (which take days to respond), innate cells act within hours, providing antigen presentation and cytokine signals that instruct T and B cells. They orchestrate phagocytosis, antigen presentation and cytokine modulation to balance maternal–foetal homeostasis. In mice, maternal macrophages cross the placenta by gestational day 7, displaying a mature phenotype that phagocytoses pre-teratogenic debris, apoptotic bodies and cellular remnants in the mesodermal layer before embryonic precursors take over at day 9 (Ref. 144). These cells, together with monocytes, engraft in foetal bone marrow as c-kit^+^ HSCs, sustaining microchimerism in peripheral leucocytes into adulthood, evident in 50% of mice where maternal effectors derive from bone marrow-resident progenitors (Ref. 129). Milk-derived macrophages (30%–47% of leucocytes) express CD14, CD11b, CD40, CD86 and HLA-DR for antigen presentation. Upon phagocytosing milk lipids, they spontaneously produce GM-CSF, which – combined with IL-4 drives differentiation into DCs capable of T-cell stimulation (Refs 145, 146). This phagocytic activity generates lipid vacuoles, altering morphology and enhancing innate surveillance. NK cells (CD56^+^/CD16^+^), a minor milk population and neutrophils (40%–60% of milk leucocytes) provide rapid cytotoxicity and mucosal barrier defence, compensating for neonatal functional immaturity (Ref. 128).

Human milk contains low concentrations of CC chemokines – RANTES (CCL5), MIP-1α (CCL3), MIP-1β (CCL4) and MCP-1/CCL2, throughout lactation (Refs 73, 147). CCL2 peaks in the first 3 months, then drops sharply. Despite low levels, RANTES promotes leucocyte infiltration and T-cell trafficking during inflammation (Refs 73, 147). Milk’s immunomodulatory environment also contains T cell-derived cytokines, including IFN-γ, TNF-α, IL-2, IL-4, IL-10 and TGF-β (Ref. 148). IL-6 is the most abundant colostrum cytokine, exerting pro-inflammatory effects (Ref. 149). IL-8 (neutrophil chemotactic factor) facilitates leucocyte migration into milk, highest in colostrum, then declining with lactation (Ref. 148). Bacteria such as Lactobacilli can partially release TNF-α from the large amounts found in colostrum (Refs 148, 150). The aqueous phase of milk contains significant IL-10, which inhibits antigen-presenting cell (APC) functions by lowering MHC II expression (Ref. 151). Breast milk increases CCL20 secretion by oral keratinocytes in the first two postnatal weeks. Given CCL20’s role in mucosal lymphoid tissue formation, this chemokine likely contributes to MALT development in newborns (Ref. 152).

Milk cytokines and soluble receptors (IL-1RA, sCD14, sTNF-RI, sTNF-RII, IL-6R and sTLR2) originate from immune cells (e.g., T cells), mammary epithelial cells, or direct maternal circulation transport (Refs 148, 149). These receptors regulate cytokines, limit gut inflammation and modulate mucosal tolerance signalling in neonates (Refs 148, 149). The effects of milk IL-5 and IL-13 on newborn T cells remain unclear (Ref. 149). Milk contains G-CSF, GM-CSF and M-CSF, though serum M-CSF is 10–100× higher (Refs 149, 153). Neonatal blood has higher GM-CSF/G-CSF than milk, yet neonatal T cells produce less GM-CSF than neonatal monocytes and undetectable G-CSF/M-CSF, collectively protecting neonates from infection.

During breastfeeding, reproductive hormones regulate T-cell recruitment, proliferation and inhibition. Prolactin, abundant in milk, drives T-cell recruitment to the mammary gland, T-cell proliferation and IFN-γ/CD80/CD86 upregulation (Refs 154, 155). Oestrogen and progesterone also modulate immunity: oestrogen regulates T-cell migration via chemokine/receptor expression, while progesterone inhibits endometrial CD8^+^ T cell cytotoxicity and dampens CD4^+^ T cell stimulation (Ref. 155).

Innate responses to microchimeric cells parallel transplantation: direct macrophage phagocytosis and indirect DC antigen presentation. During offspring infections, milk anti-inflammatory macrophages (CD16^−^ˡ°ʷ) predominate, increasing leucocytes and IL-6/IL-8 to recruit more cells via adhesion/diapedesis in GALT, promoting tolerance to emerging microbiota (Refs 142, 156). Milk DC-SIGN (C-type lectin) mediates pathogen interactions (e.g., HIV binding) – potentially raising vertical transmission risk but also priming neonatal adaptive responses (Refs 142, 157). Milk neutrophils serve as the neonatal frontline against mucosal pathogens; their short lifespan limits persistence but enables immediate opsonization via transferred IgG (Ref. 158). In foetal tissues, maternal monocytes differentiate into tissue macrophages that express TGF-β to induce NIMA-specific Tregs, suppressing effector T-cell lymphoproliferation and hypersensitivity (Refs 34, 80, 159). Breastfeeding reinforces this: oral NIMA exposure via milk maintains higher Treg/effector ratios as maternal innate cells migrate transepithelially through loose neonatal tight junctions to lymphoid organs (Ref. 80). In immunodeficient foetuses, engraftment rates rise (e.g., 86% thymus colonization by maternal macrophages/NK cells), underscoring innate cells’ role in opportunistic infection defence (Ref. 78).

In recurrent miscarriage, innate dysregulation amplifies trafficking. Placental dysfunction (pre-eclampsia, trisomy 21) elevates foetal monocyte/macrophage passage, correlating with preterm labour and increased cffDNA, as activated macrophages drive IL-6/TNF-α inflammation (Refs 93, 160). Uterine NK cell mismatch may trigger trophoblast cytotoxicity via perforin/granzyme, worsening secondary recurrent miscarriage (Ref. 161). Protective innate priming from prior pregnancies (e.g., milk neutrophils strengthening mucosal barriers) reduces pre-eclampsia recurrence with the same partner (Ref. 162). However, elevated neutrophils (especially in the second trimester) are associated with pre-eclampsia (Refs 163, 164). A critical unresolved question is whether elevated neutrophils cause or the consequence? Neutrophil elevation precedes symptoms in some studies, suggesting causality, but interventional trials (e.g., colchicine) in at-risk pregnancies are lacking and ethically challenging.

In autoimmune diseases, foetal innate microchimerism contributes to GVHD-like pathology. In SSc, foetal macrophages accumulate in skin lesions, displaying Th2-biased (IL-4/IL-13) profiles that promote fibrosis via TGF-β, more prevalent in inflamed tissues than in blood (Ref. 92). Variability reflects subtypes: foetal DCs in limited cutaneous SSc drive autoantibody diversification via HLA-DR presentation (Ref. 165). In PBC, Y-chromosome-positive macrophages in liver biopsies (8/19 patients) correlate with injury, though findings are inconsistent across studies (Ref. 139). In Sjögren’s syndrome, foetal monocytes appear in salivary lesions (not blood), suggesting tissue-specific chemokine recruitment (Ref. 166). In Hashimoto’s/Graves’ thyroiditis, foetal NK cells/monocytes increase in affected glands (83% vs. 75% of controls), potentially via HLA-DQA1*0501 susceptibility alleles that fuel autoimmunity (Ref. 167). In JIIM, maternal innate cells (e.g., monocyte-derived macrophages) in muscle lesions exacerbate inflammation. In NLS heart block, they express alpha-actin for reparative modulation. In preterm lungs, expanded foetal-activated macrophages and Tbet^+^ B cells induce airway hyperresponsiveness and goblet cell hyperplasia via IL-6/TNF-α, contrasting with term birth’s CD4^+^ dominance (Ref. 15).

In cancer, innate cells play dual surveillance/angiogenic roles. In high-grade breast tumours, foetal macrophages express non-tumoral epithelial markers, aiding stromal repair but potentially fuelling progression via VEGF (Ref. 120). Parous women with foetal monocyte microchimerism have lower breast/ovarian cancer risk via enhanced phagocytosis/complement (Ref. 168). In murine melanoma models, foetal NK progenitors home to tumours, potentially suppressing growth via perforin, though DC presentation may inadvertently promote angiogenesis (Ref. 108). Skin tumours show 75% FMc-positive stroma with monocyte-derived endothelial markers (Ref. 109). In thyroid cancer, foetal macrophages (CD45^+^/MHC II^−^) destroy tumour cells, while Tg^+^ subsets repair tissue (Ref. 113). Overall, innate microchimerism reduces malignancy in the bladder, brain, pancreas, colorectal, lymphoma and leukaemia via immunosurveillance.

In tissue repair, innate cells drive regeneration. In animal models, foetal macrophages recruit to maternal liver/kidney injury postdelivery, phagocytosing debris and secreting growth factors for resolution (Ref. 116). In brain trauma, they cross the BBB and differentiate into microglia-like cells (Ref. 117). Pregnancy appendicitis shows elevated male neutrophils at inflamed sites (Ref. 110). Late-pregnancy myocardial infarction recovery is high, with foetal monocyte progenitors homing to infarcted tissue and differentiating into connexin 43/troponin T^+^ cardiomyocytes (Refs 120, 121). Wounded skin recruits Ccl2^+^ foetal myeloid progenitors that transdifferentiate into endothelial cells for neovascularization; exogenous Ccl2 accelerates healing only in postpartum mice (Ref. 38). In fibrotic wounds, persistent macrophages delay remodelling (Ref. 38). Milk neutrophils/monocytes neutralize perinatal pathogens, maturing foetal innate responses (Ref. 169).

Immune transfer to offspring through milk and maternal innate cells bolsters neonatal defences. Macrophages/DCs migrate to GALT, presenting antigens via CD83/DC-SIGN to prime T/B responses, with GM-CSF enabling DC maturation (Ref. 145). During offspring respiratory infections, milk leucocyte influx (macrophages ↑, pro-inflammatory ↓) via IL-8 adhesion protects the mucosa (Ref. 170). NK/neutrophils compensate for cytotoxicity gaps, persisting in lungs/heart (63%–86% engraftment) (Ref. 171). In the Nb model, maternal monocyte-derived macrophages activate innate effectors and transfer Th2 memory (Ref. 125). Milk macrophage TGF-β induces neonatal Treg anergy, suppressing anti-NIMA responses (Ref. 72). Stem-like monocytes (CD34^+^CD133^−^) differentiate into myeloid progenitors expressing SOX2/NANOG for multilineage support (Refs 143, 170). In summary, innate immune cells in foetomaternal microchimerism weave phagocytosis, antigen presentation and homing networks from debris clearance (GM-CSF/TGF-β) to dual repair/surveillance.

### Foetomaternal microchimerism and HLA Alleles

All cellular interactions described above, Tregs, CTLs, B cells and innate cells, are governed by HLA compatibility. HLA class I/II mismatches determine allorecognition strength, shape molecular dialogue by presenting peptides to T cells and engage complement to amplify or dampen inflammation. Together, HLA and complement serve as critical sentinels in foetomaternal microchimerism, modulating allorecognition, tolerance induction and effector responses while enabling immune memory transfer (Refs 172, 173). HLA class I/II mismatches drive maternal–foetal trafficking. Soluble HLA (sHLA) and membrane-bound forms cross the placenta and breast milk, priming NIMA-specific tolerance in offspring (Refs 65, 174). HLA-DRB1 polymorphisms distinguish IPA from NIMA, enabling non-invasive prenatal testing via cffDNA, which comprises 1.4%–5.4% of maternal plasma (Ref. 175). Complement components (C3, C5a) amplify innate responses by opsonizing microchimeric cells for phagocytosis, while C1q promotes decidual NK tolerance via trophoblast HLA-G expression (Ref. 176). These interactions intensify in placental dysfunction (e.g., trisomy 21, pre-eclampsia), increasing foetal cell passage sixfold and correlating with elevated complement activation markers (Ref. 93).

HLA orchestrates direct and indirect allorecognition in microchimerism, similar to graft surveillance. In utero, maternal HLA exposes foetal T cells to NIMA, inducing CD4^+^CD25ʰⁱᵍʰFOXP3^+^ Tregs via TGF-β-dependent pathways in lymph nodes, suppressing anti-maternal effector responses and establishing lifelong tolerance (Refs 34, 177). Postnatally, breastfeeding-delivered sHLA sustains this by generating additional NIMA-specific Tregs that outpace effector T cells, correlating with reduced hypersensitivity (Refs 178, 179). Complement tags discordant cells with C3b for macrophage clearance, while foetal HLA-E inhibits membrane attack complex (MAC) formation to avoid lysis (Refs 180, 181). In rhesus monkeys and mice, HLA-compatible transfers (e.g., maternal HLA-DQB1*0301) boost engraftment frequencies, favouring higher microchimerism in compatible tissues without MHC II recognition (Ref. 182). Dysregulation, for example, HLA class II presenting Y-epitopes in secondary miscarriage, amplifies complement-mediated trophoblast damage via C5a-induced inflammation (Ref. 183).

In recurrent miscarriage, HLA mismatches trigger alloantibody and complement cascades. Secondary recurrent miscarriage after a successful pregnancy is associated with HLA class II (e.g., DQA1*0501, DQB1*0201/0301) presenting paternal antigens, activating complement C3 convertase and MAC, reducing live birth likelihood after a male firstborn (Refs 98, 184). Protective NIMA-matching in renal grafts enhances survival via complement inhibition and Treg expansion, outperforming IPA donors (Ref. 185). However, the clinical utility of HLA typing in recurrent miscarriage remains controversial (most studies are retrospective/underpowered). A prospective multicentre trial stratifying by HLA class II compatibility with the male partner is urgently needed. Until then, HLA typing for secondary recurrent miscarriage should be considered experimental, not standard of care.

In autoimmune diseases, HLA-driven microchimerism mimics chronic GVHD. In SSc, increased HLA class II compatibility between patients and offspring elevates foetal cell engraftment, minimizing alloreactivity and promoting persistence in PBMCs and skin lesions (Ref. 101). Complement C3 deposition in lesions amplifies fibrosis; foetal cells appear in 17/19 SSc cases versus controls (Refs 102, 186). In PBC, HLA-linked Y-chromosome-positive cells in the liver (8/19) correlate with complement activation, though findings vary (Ref. 139). In Sjögren’s syndrome, HLA facilitates foetal monocyte accumulation in salivary glands, with C1q shielding from clearance (Ref. 89). Hashimoto’s/Graves’ thyroiditis features HLA-susceptible alleles that boost foetal NK/complement interactions in glands (83% vs. 75%) (Refs 98, 187). In JIIM/NLS, maternal HLA-microchimerism in muscle/heart triggers complement-mediated block via anti-Ro/La IgG (Refs 188, 189). Preterm lungs show HLA-mismatched foetal macrophages driving C5a/IL-6 inflammation and hyperresponsiveness (Ref. 190).

In cancer, HLA/complement duality aids surveillance. Foetal HLA-G on microchimeric cells evades complement lysis, enabling stroma infiltration in breast carcinomas while priming IgG for ADCC against HA-1 antigens (Refs 85, 106). Parous women with HLA-compatible FMC show reduced breast/ovarian risk via complement opsonization of tumours (Refs 191, 192). In melanoma, HLA-E+ foetal progenitors form VEGF-recruited vessels, but complement C3 limits spread (Ref. 108). Skin tumours (75% FMc+) involve HLA-presenting DCs in stroma, with C1q modulating angiogenesis (Ref. 108). Protective in bladder/brain/pancreas cancers via HLA-restricted cytotoxicity (Refs 193, 194); thyroid favours repair (Ref. 113).

In tissue repair, HLA/complement guides homing. Foetal HLA-mismatched cells recruit via C5a gradients to injured liver/kidney/brain, where C3b opsonization aids macrophage phagocytosis (Refs 115, 116). Appendicitis elevates complement at sites of inflammation (Ref. 110). Myocardial infarction recovery leverages HLA-G-shielded progenitors differentiating amid C3-modulated neoangiogenesis (Ref. 38). Skin wounds use CCL2/complement for myeloid recruitment, with HLA-DR+ DCs presenting for resolution (Ref. 38).

In immune transfer, milk sHLA/complement primes neonatal tolerance. HLA exposure via breastfeeding sustains NIMA-Tregs, with C3 complementing IgG opsonization against pathogens (Refs 179, 195). In the Nb model, HLA-presented antigens from milk DCs activate innate effectors (Ref. 125). TGF-β/HLA synergy induces anergy (Refs 72, 133). In summary, HLA and complement forge allorecognition networks in foetomaternal microchimerism from NIMA tolerance (sHLA/TGF-β) to effector clearance (C3/MAC). These axes evolutionarily underpin health, informing therapies for HLA-linked autoimmunity, miscarriage and cancer.

### Foetomaternal microchimerism and MSCs and their EVs

Beyond traditional immune cells, extracellular vehicles (EVs), exosomes and microvesicles and MSCs act as potent non-cellular mediators in foetomaternal microchimerism, enabling signal transfer and multilineage regeneration (Ref. 51). EVs from trophoblasts and foetal progenitors shuttle miRNAs (e.g., miR-146a, anti-inflammatory) and proteins across the placenta, shifting maternal T cells towards Th2 tolerance and suppressing complement via C1q inhibition (Ref. 196). EV-borne miR-146a/miR-155 suppress maternal NF-κB and IFN-γ pathways, while MSC-derived IDO depletes tryptophan to arrest effector T-cell proliferation. EV-embedded HLA-G and TGF-β mimic cellular microchimerism, priming NIMA-specific Tregs without direct engraftment as seen in rhesus monkeys where EV transfer parallels CD34^+^ HSC persistence for up to 6 months postpartum (Ref. 197). MSCs, detected in first-trimester foetal circulation and maternal blood post-termination, express VEGFR2/CD31 for endothelial differentiation, contribute to decidual neoangiogenesis and evade allorejection via low MHC II expression (Refs 109, 198). These ‘pregnancy-associated progenitor cells’ (PAPCs) engraft in maternal lungs and heart, with inflammation-linked trafficking amplified in pre-eclampsia (Ref. 93).

EVs and MSCs orchestrate indirect communication in microchimerism, extending transplantation-like tolerance. In milk, EVs carry soluble HLA and cytokines (IL-10, TGF-β), reinforcing postnatal NIMA exposure and Treg expansion via Smad signalling, outpacing effector responses in GALT (Ref. 65). Molecularly, exosomal miR-155 downregulates maternal IFN-γ, dampening Th1-driven inflammation, while MSC-secreted IDO inhibits T-cell proliferation (Ref. 199). In mice, GFP-labelled foetal EVs home to maternal injury sites, fusing with resident cells for hybrid phenotypes that enhance phagocytosis without GVHD risk (Ref. 200). Post-breastfeeding, EV-mediated transfer sustains microchimerism niches, with HLA-DQB1 compatibility boosting EV uptake in foetal lymph nodes (Ref. 182).

In recurrent miscarriage, dysregulation of EVs and MSCs worsens alloimmunity. Reduced EV-HLA-G in secondary miscarriage correlates with complement C5a surge and trophoblast lysis, while trisomy 21 elevates MSC trafficking sixfold, linking to preterm risks (Refs 160, 201). Protective prior-pregnancy EVs reduce pre-eclampsia via miRNA-mediated vascular repair (Ref. 162).

In autoimmune diseases, EVs and MSCs contribute to chronicity. In SSc, foetal EV-miRNAs in lesions promote fibrosis via TGF-β amplification, with MSCs in PBMCs/skin (17/19 cases) differentiating into myofibroblasts (Refs 92, 95, 102, 165, 186). PBC shows EV-embedded Y-DNA in liver (8/19), variably activating complement (Ref. 139). Sjögren’s syndrome features MSC homing to salivary glands via chemokine gradients (Ref. 166). Thyroiditis involves HLA-susceptible EV transfer boosting NK/complement in glands. JIIM/NLS shows maternal MSCs in muscle/heart, modulating via alpha-actin but triggering blocks (Ref. 98).

In cancer, EVs and MSCs have dual roles. Protective EV-miR-146a suppresses breast tumours, reducing risk in parous women (Ref. 196); MSCs in high-grade stroma express HLA-G for immune evasion but aid immunosurveillance via HA-1 presentation (Ref. 106). EV-recruited MSCs protect against ovarian, brain and leukaemic cancers via complement opsonization (Ref. 193), while thyroid favours repair (Ref. 113). A critical caveat: most EV studies use in vitro systems or bolus injections, neither of which recapitulates continuous, low-level pregnancy EV release. EV cargo also varies dramatically with gestational age and maternal health; ‘protective’ EVs in a healthy pregnancy may become ‘pathogenic’ in inflammation. Single-EV profiling is needed to resolve this heterogeneity.

In tissue repair, EVs/MSCs excel in homing. Foetal EVs transport growth factors to liver and kidney injuries for resolution (Ref. 202); brain trauma: EV-miRNAs cross the BBB for microglial modulation (Ref. 117). Appendicitis: elevated EVs during inflammation (Ref. 110). Myocardial infarction: MSC-EVs improve contractility via connexin (Refs 120, 121). Skin wounds: CCL2-recruited MSC-EVs drive neovascularization, stage-specific (Ref. 38). In immune transfer, milk EVs/MSCs prime neonates. EVs carry sHLA/TGF-β for Treg anergy (Ref. 203). Pluripotent MSC-like EVs (OCT4/NANOG+) differentiate multilineage without tumours (Ref. 204). In summary, EVs and MSCs in foetomaternal microchimerism enable vesicle-mediated signalling networks from miRNA tolerance to progenitor repair; axes like HLA-G/TGF-β evolutionarily enhance health, targeting exosome therapies for miscarriage, autoimmunity and regeneration.

## Temporal and phenotypic evolution of microchimerism during gestation

The immunological impact of microchimerism is fundamentally shaped by the timing of transfer and the evolving phenotype of chimeric cells across trimesters. While early gestation focuses on establishing baseline allotolerance through primitive progenitors, late pregnancy shifts towards systemic imprinting and tissue repair via mature effector populations. This trimester-specific gradient, transitioning from stem-like to effector-like phenotypes, is summarized in Table 2.

## Association between prior pregnancy number (parity) and transferred microchimerism or its immune function

**Figure 2.**
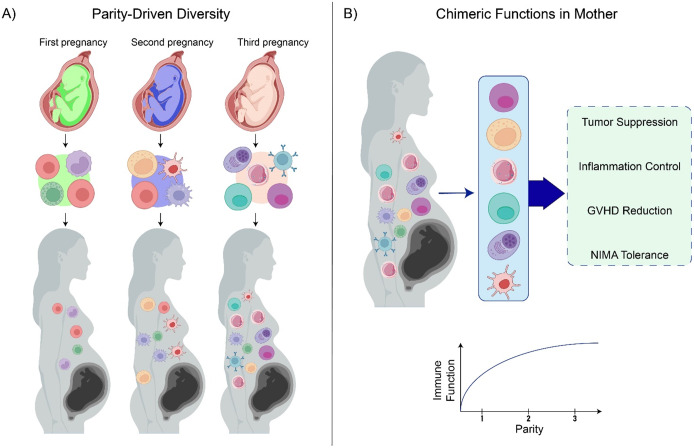
Increasing parity (A) expands chimeric functional diversity, including anti-cancer, anti-inflammatory, graft protection and foetal tolerance without a linear increase in cell number (B). Figure 2. long description.

Increasing parity reshapes the maternal chimeric landscape, not through simple additive accrual but via dynamic displacement and heterogeneity, broadening functional repertoires and supporting immune homeostasis (Figure 2) (Refs 35, 45). FMc burden does not scale linearly with parity. Instead, functional diversification across pregnancies may underpin improved maternal outcomes. For example, increased Treg-mediated Th1 suppression has been observed in some women with rheumatoid arthritis (Refs 4, 45). At the molecular level, repeated foetal exposure may expand NIMA tolerance repertoires. Enduring chimeric cells (persisting decades in marrow or organs) engage in ‘graft-within-graft’ interactions – potentially mitigating GVHD risk while enhancing graft-versus-tumour efficacy via minor antigen priming (e.g., HA-1) (Refs 35, 54). MMc often declines with increasing parity, possibly due to deletion or replacement by new FMc, but this decline may benefit foetal tolerance in the next generation. In transplantation studies, multiple donors have shown reduced leukaemia relapse, associated with enhanced humoral protection (Ref. 45). Parity-dependent chimeric variation correlates with reduced risk of certain cancers (e.g., breast, ovarian) via increased IgG/complement opsonization and EV-encapsulated miRNA transport. Conversely, in autoimmune diseases (e.g., JIIM/SLE), MMc accumulation associates with tissue-specific inflammation (Refs. 18, 37). Thus, parity enriches chimeric functional diversity, protective in the mother, regulatory in the offspring, without a linear increase in number, suggesting an ‘evolutionary equation’ between maternal advantage and generational cost (Refs. 13, 168).

## Niche-specific modulation: the role of the microenvironment

The functional fate of microchimeric cells is not fixed; it is dynamically recalibrated by the surrounding tissue microenvironment. Cytokine gradients, oxygen levels and extracellular matrix (ECM) signals dictate whether these cells adopt a reparative or a pro-inflammatory role. This tissue-specific duality, where parenchymal organs often favour integration while mucosal or injured tissues may trigger inflammatory responses, is detailed in Table 3.

**Table 3.** Impact of tissue-specific microenvironments on chimeric cell fate Table 3. long description.

Organ/niche	Microenvironmental signals	Chimeric cell transformation	Functional impact	References
Brain	Low O_2_, lack of inflammation	Microglia-like (MMc)	Neuroimmune regulation (↑IL–10, low IFN-γ)	(Refs 4, 54, 117)
Liver	C3b opsonization, repair cues	Hybrid hepatocytes (CK^+^)	Parenchymal repair and regeneration	(Refs 35, 54, 116, 205, 206)
Heart	C5a gradients, connexin 43	MSC-like (FMc/Cdx2^+^)	Post-MI reparative integration	(Refs 120, 121)
Lung	Preterm inflammatory signals	Activated macrophages (IL–6, TNF-α)	Mucosal hyperresponsiveness; goblet cell hyperplasia	(Refs 4, 15, 53)
Skin	CCL2 gradients/TGF-β	Endothelial (repair) versus profibrotic	Wound healing versus systemic sclerosis (SSc) fibrosis	(Ref 38)
Decidua	Reduced CXCL9/10, miR–146a	Myeloid progenitors	Foetomaternal tolerance; Th2 predominance	(Refs 4, 51)

## Persistent challenges in microchimerism research

Despite over two decades of active investigation since the first descriptions of foetal cell transfer during human pregnancy, the field of foetomaternal microchimerism continues to face fundamental challenges that limit both mechanistic understanding and clinical translation. These challenges are distributed across a number of domains, including technical limitations in the sensitivity of detection (current quantitative PCR (qPCR) detects male DNA at approximately 1 in 10^5^ cells, whereas true chimeric cell frequencies in healthy, parous women are estimated to be between 1 in 5 × 10^5^ and 1 in 10^7^ peripheral blood mononuclear cells), unresolved causality in human disease associations (the inability to distinguish whether microchimeric cells actively drive pathology or are passively recruited to sites of pre-existing inflammation), substantial heterogeneity in study methodologies that precludes cross-cohort comparisons and a lack of standardized clinical protocols for therapeutic applications, such as NIMA-informed donor selection in haematopoietic stem cell transplantation. The aforementioned challenges are systematically summarized in Table 4, which provides a domain-specific analysis of current limitations, key unresolved questions and actionable future directions.

**Table 4.** Challenges and future therapeutic potentials in microchimerism research Table 4. long description.

Research/clinical area	Current limitations	Key challenges	Future therapeutic/technological potential	References
Detection and characterization	Heavy reliance on bulk PCR, Y-chromosome markers and cffDNA quantification; limited cellular resolution	qPCR sensitivity: 5 × 10^−4^ to 10^−5^; FISH sensitivity ranges from 10^−2^ to 10^−6^ depending on target; detection rates of male cells in parous women vary from 30 to 50% across studies; no method captures spatial distribution of rare cell subsets	Digital PCR achieves 5 × 10^−5^ to 5 × 10^−6^ sensitivity; dPCR predicts relapse median 63 days earlier versus 45.5 days by qPCR; LoD of 0.01% for both qPCR and dPCR, LoQ of 0.1% by qPCR versus 0.05% by dPCR; single-cell analysis requires pre-enrichment and limited cell number	(Refs 94, 175, 207, 208)
Functional interpretation	Predominantly observational associations; limited causal evidence	FMc detected in healthy controls and disease groups with variable frequencies; 17/19 SSc cases versus controls; breast cancer cases show significantly lower FMc frequency than healthy women (*p* < 0.05); outlier breast cancer cases had 277 and 374 foetal genomes per 10^6^ maternal genomes versus median 2	Systems immunology approaches; organoid/placenta-immune co-culture models; prospective cohort studies with pre-conception baseline sampling needed to establish temporality	(Refs 209, 210)
Temporal dynamics and persistence	Trimester-dependent patterns described, but long-term persistence and reactivation triggers unclear	FMc persists decades postpartum; proportion of parous women with detectable male cells unknown but likely underestimated; detection rates increase with improved sensitivity; multiple pregnancy history may displace pre-existing chimeric cell populations	Real-time imaging; decade-scale cohort studies; barcoded lineage tracing in primate models; longitudinal sampling pre-conception, during pregnancy and postpartum	(Refs 13, 45, 51)
Clinical translation (HSCT and tolerance induction)	Promising NIMA/HSCT signals but no standardized clinical protocols	NIMA-mismatched donors associated with decreased aGvHD risk: HR 0.1 (95% CI 0.02–0.6, *p* = 0.001) for grade II-IV aGvHD; NIMA matching retained beneficial role for sibling, child, maternal donors and cord blood units; differences in detection methods and transplantation protocols impede cohort comparison	NIMA-aware donor selection; evidence suggests only one dominant microchimeric cell population persists at a time; studies should combine NIMA analysis with direct detection of microchimeric cells pre-HSCT	(Refs 45, 50, 192)
Autoimmunity versus oncology (dual roles)	Conflicting evidence: protective anti-tumour surveillance versus association with autoimmune lesions	Breast cancer: parous women have a lower risk (OR 0.72); FMc significantly less frequent in breast cancer patients than healthy controls; proposed mechanism: allogeneic foetal cells provide immune surveillance; microchimerism-positive cancer patients showed higher survival and response rates with haplo-PBSC treatment	Foetal cells with multilineage potential identified in maternal tissues with various pathologies, suggesting a stem cell-like response to tissue damage; therapeutic potential of allogeneic foetal cell immune surveillance; outlier cases with high FMc burden (277–374 genomes/10^6^) suggest a rare possibility of foetal cell transformation	(Refs 92, 104, 106, 108, 139)
Maternal–neonatal interface (milk, EVs, MSCs)	Incomplete cargo mapping of milk/placental EVs and cellular components	Breastfeeding route of NIMA exposure; relationship between oral tolerance and systemic microchimerism maintenance	Therapeutic exploitation of breast-milk exosomes for neonatal immune priming; engineered EVs for tolerance induction	(Refs 51, 203, 204)
Ethical, regulatory and translational implications	Cross-generational cell transfer raises identity/consent questions; regulatory frameworks absent	FMc persists without consent from offspring; potential germline implications of manipulating microchimeric cells	Development of ethical frameworks, regulatory guidance and translational pipelines integrating long-term follow-up	(Refs 54, 168)

## Conclusion and discussion

This review reconceptualizes foetomaternal microchimerism as an active biological dialogue rather than a passive remnant of pregnancy, connecting generations through sustained cellular and molecular exchange. The findings demonstrate that pregnancy establishes a distinct immunological environment in which self and non-self-cooperate rather than oppose. FMc assimilate into maternal organs, inducing FOXP3-positive regulatory T cells, activating CTLA-4 and promoting the production of IL-10 and TGF-β. Conversely, maternal microchimeric cells populate the foetal thymus and lymphoid organs, shaping non-inherited maternal antigen tolerance through PD-L1-positive dendritic cell pathways. Together, these reciprocal interactions form a tolerance continuum in which maternal and foetal immune systems coevolve towards equilibrium, creating a biological memory that persists for decades after gestation.

A central paradox emerges from these observations. Identical cells that maintain tolerance and tissue integrity can, under specific genetic or inflammatory conditions, become pathogenic. Foetal microchimerism accelerates wound healing, improves cardiac repair and enhances cancer surveillance, yet its persistence in autoimmune-prone environments exacerbates fibrosis and antigen mimicry, as observed in scleroderma and primary biliary cholangitis. Similarly, maternal cells that protect neonates may, in rare circumstances, predispose offspring to autoimmunity. These dualities position microchimerism as a dynamic equilibrium between regeneration and autoaggression, representing an evolutionary trade-off in which maternal immunological clarity is partially sacrificed for reproductive success.

Despite the conceptual scope of this framework, the field faces substantial methodological constraints. Current detection methods, including polymerase chain reaction and short-read sequencing, cannot resolve the phenotypic heterogeneity, epigenetic flexibility, or spatiotemporal fate of microchimeric cells. Longitudinal human studies remain limited, and causal relationships are poorly understood. Microchimerism is neither inherently pathogenic nor protective; its net effect depends on host genetics, the local microenvironment and timing. This complexity underscores the need for longitudinal multiomic studies capable of distinguishing causality from correlation.

Three grand challenges must be addressed before clinical translation becomes feasible. First, non-invasive methods for real-time chimeric cell tracking without biopsy are required. Second, causal links between specific chimeric cell subsets and disease outcomes must be established using lineage tracing models. Third, ethical and technical barriers to human interventional studies that manipulate microchimerism, whether by enhancing regulatory T-cell expansion or depleting pathogenic clones, need to be overcome. Success in these areas demands interdisciplinary collaboration among reproductive immunologists, bioengineers and clinical trialists.

Future investigations should employ single-cell multiomics, high-resolution lineage tracking and in situ imaging to map the life cycles of chimeric cells over decades. Interventional strategies, including engineered regulatory T-cell expansion, modulation of exosomal signalling, or selective HLA matching, could offer novel therapeutic opportunities in transplantation, autoimmunity and regenerative medicine. Collectively, foetomaternal microchimerism reveals that immunity is not an isolated defence mechanism but a hereditary continuum, a dynamic connection through which each generation contributes cellularly and molecularly to the next.

## Data Availability

No data were used for the research described in the article.

## Abbreviations

aGvHD: acute graft-versus-host disease
APC: antigen-presenting cell
BBB: blood–brain barrier
C1q, C3, C5a, MAC: complement components
CCL2 (MCP-1): chemokine ligand 2
CCR2: chemokine receptor 2
CD34, CD45, CD103: cluster of differentiation markers
cffDNA: cell-free foetal DNA
CNS1: conserved non-coding sequence 1
CTL: cytotoxic T lymphocyte
CTLA-4: cytotoxic T-lymphocyte-associated protein 4
DC: dendritic cell
EV: extracellular vesicle
FMc: foetal microchimerism
FOXP3: forkhead box protein P3
GALT: gut-associated lymphoid tissue
GVHD: graft-versus-host disease
GvL: graft-versus-leukaemia
HA-1: minor histocompatibility antigen 1
HLA: human leucocyte antigen
HSC: haematopoietic stem cell
HSCT: haematopoietic stem cell transplantation
HY: Y-chromosome antigen
IDO: indoleamine 2,3-dioxygenase
IFN-γ: interferon-gamma
IL: interleukin
IPA: inherited paternal antigen
JIIM: juvenile idiopathic inflammatory myopathy
MMc: maternal microchimerism
MSC: mesenchymal stromal cell
NF-κB: nuclear factor kappa-B
NIMA: non-inherited maternal antigen
NK: natural killer cell
NLS: neonatal lupus syndrome
PBC: primary biliary cholangitis
PBMC: peripheral blood mononuclear cell
PD-1: programmed cell death protein 1
PD-L1: programmed death-ligand 1
RA: rheumatoid arthritis
SLE: systemic lupus erythematosus
Smad: mothers against decapentaplegic homolog
SRY: sex-determining region Y
SSc: systemic sclerosis (scleroderma)
TGF-β: transforming growth factor beta
Th: T helper cell
TLR: toll-like receptor
TNF-α: tumour necrosis factor alpha
Treg: regulatory T cell
VEGF: vascular endothelial growth factor

## Long descriptions

### Figure 1. Long description

The diagram is divided into two main vertical sections representing different gestational stages and outcomes.

At the top left, a silhouette of a pregnant woman at gestational week 7 shows red microchimerism (F M c) trafficking from the fetus to the mother. A circular callout displays a cluster of cells including N K, B cell, P C, M Q, Memory C D 8 plus, Memory C D 4 plus, and C D 38 plus / C D 34 plus. Below this, three vertical paths lead to outcomes. The first path shows Fetal-H S C developing into various immune cells, leading to the development and enhancement of the infant’s immune system. The second path involves Memory C D 4 plus and C D 8 plus T cells, T regs, and Dendritic cells (P D-L 1 plus), leading to increased N I M A T reg. The third path involves P D-L 1, T G F-beta, I L-10, and C C L 2 / C C R 2.

At the top right, a silhouette at week 13 shows green microchimerism (M M c) trafficking from the mother to the fetus. The circular callout includes M Q, T h-2, M S C, D C, C T L, N K, Monocyte, and C D 38 plus / C D 34 plus. Below this, three vertical paths lead to different outcomes. The first path involves M S C, E V s, and myeloid progenitors, leading to suppressed inflammation. The second path involves T h 2-polarized C D 4 plus T cells, I L-10, T G F-beta, and C T L A-4, leading to increased T reg expansion and function. The third path involves M S C and m i R N A / V E G F R 2, leading to tissue repair and angiogenesis, with a dashed line pointing to increased cancer risk.

Navigate back to Figure 1..

### Table 1. Long description

The table consists of seven columns: Molecular pathway, Key cytokines/molecules, Primary cellular source, Biological source (maternal/foetal origin), Functional effect, Immunological interpretation, and References.

* T G F-beta/Smad axis: Includes T G F-beta, Smad 2/3, and Foxp3 from Tregs and decidual macrophages. Predominantly maternal origin. Induces regulatory T cells and inhibits effector Th 1/Th 17 activity to maintain pregnancy tolerance.

* I L-10/C T L A-4 pathway: Includes I L-10, C T L A-4, and I D O from maternal D Cs and F O X P 3 super + T cells. Maternal origin. Suppresses antigen presentation to prevent foetal rejection.

* Th 1/Th 2/Th 17 polarization: Includes I F N-gamma, I L-4, I L-13, and I L-17 from C D 4 super + T helper subsets. Shared maternal and foetal origin. Th 2 dominance promotes tolerance while Th 1/Th 17 drives pathology.

* Chemokine signalling: Includes C C L 2/C C R 2, C X C L 9/10, and C X C R 3 from foetal myeloid progenitors and maternal leucocytes. Bidirectional origin. Guides cell trafficking to direct the spatial dialogue between tolerance and inflammation.

* P D-L 1/P D-1 checkpoint: Includes P D-L 1, P D-1, and C D 80/86 from D Cs, trophoblasts, and T cells. Dominant foetal trophoblast expression. Inhibits T-cell activation to recreate immune silence.

* H L A-G/Complement system: Includes H L A-G, C 3, and C 5 a from trophoblasts and macrophages. Primarily foetal origin. Blocks complement-mediated lysis and promotes N K cell tolerance.

* E V-mediated mi R N A transfer: Includes mi R-146 a, mi R-155, and T G F-beta cargo from M S Cs and placental E Vs. Predominantly foetal origin. Epigenetic suppression of N F-kappa B for intercellular immune education.

Navigate back to Table 1..

### Table 2. Long description

The table consists of six columns and three data rows organized by trimester.

* Header Row: Trimester, F M c frequency/burden, Dominant phenotypes, Key molecular drivers, Immunological outcome, References.

* First Trimester Row:

- F M c frequency/burden: Detectable (4 to 6 weeks).

- Dominant phenotypes: C D 34 super plus, C D 38 super plus (stem-like).

- Key molecular drivers: T G F-beta/Smad, S O X 2, N A N O G.

- Immunological outcome: Central/peripheral tolerance; N I M A-specific T regs.

- References: Refs 4, 51, 52.

* Second Trimester Row:

- F M c frequency/burden: approximately 1 per 1,000 P B M C s.

- Dominant phenotypes: V E G F R 2 super plus, C D 31 super plus (mesenchymal).

- Key molecular drivers: Th 2-cytokines, mi R N A-E V s.

- Immunological outcome: Thymic education; diversification; pregnancy support.

- References: Refs 4, 45.

* Third Trimester Row:

- F M c frequency/burden: Peak (greater than 100 cells/m L).

- Dominant phenotypes: Mature Effectors (N K, macrophages).

- Key molecular drivers: mi R N A-loaded E V s, C f f D N A.

- Immunological outcome: Systemic imprinting; postpartum repair; transgenerational benefits.

- References: Refs 51, 53, 54.

Navigate back to Table 2..

### Figure 2. Long description

Panel A, titled Parity-Driven Diversity, shows three vertical columns for First pregnancy, Second pregnancy, and Third pregnancy. At the top, fetuses are colored green, blue, and pink respectively. Arrows point down to clusters of immune cells. The first pregnancy shows four cells, the second shows five, and the third shows six distinct cell types. Further arrows point to silhouettes of pregnant mothers, showing an accumulation of these diverse colored cells within the maternal body over time. Panel B, titled Chimeric Functions in Mother, features a silhouette of a pregnant woman on the left with various colored chimeric cells distributed throughout her body. An arrow points from the silhouette to a vertical pill-shaped container holding six different cell types. A large blue arrow points from this container to a green box listing four benefits: Tumor Suppression, Inflammation Control, G V H D Reduction, and N I M A Tolerance. Below this is a line graph where the x-axis is Parity (numbered 1, 2, 3) and the y-axis is Immune Function. The data shows an asymptotic curve that rises sharply from parity 0 to 1 and then gradually levels off as parity increases to 3.

Navigate back to Figure 2..

### Table 3. Long description

The table consists of five columns and six rows of data.

Row 1: Brain. Signals include low O sub 2 and lack of inflammation. Transformation results in Microglia-like M M c cells. Functional impact is neuroimmune regulation with increased I L 10 and low I F N gamma.

Row 2: Liver. Signals include C 3 b opsonization and repair cues. Transformation results in Hybrid hepatocytes C K plus. Functional impact is parenchymal repair and regeneration.

Row 3: Heart. Signals include C 5 a gradients and connexin 43. Transformation results in M S C-like F M c or C d x 2 plus cells. Functional impact is post-M I reparative integration.

Row 4: Lung. Signals include preterm inflammatory signals. Transformation results in activated macrophages producing I L 6 and T N F alpha. Functional impact is mucosal hyperresponsiveness and goblet cell hyperplasia.

Row 5: Skin. Signals include C C L 2 gradients or T G F beta. Transformation results in endothelial repair versus profibrotic cells. Functional impact is wound healing versus systemic sclerosis S S c fibrosis.

Row 6: Decidua. Signals include reduced C X C L 9 or 10 and m i R 146 a. Transformation results in myeloid progenitors. Functional impact is foetomaternal tolerance and T h 2 predominance.

Navigate back to Table 3..

### Table 4. Long description

The table consists of five columns and seven rows of data.

Row 1: Detection and characterization. Limitations include reliance on bulk P C R and Y-chromosome markers. Challenges include q P C R sensitivity of 5 times 10 super minus 4 to 10 super minus 5. Future potential includes digital P C R achieving 5 times 10 super minus 5 to 5 times 10 super minus 6 sensitivity.

Row 2: Functional interpretation. Limitations include observational associations. Challenges include F M c detection in 17 out of 19 S S c cases. Future potential includes systems immunology and organoid co-culture models.

Row 3: Temporal dynamics and persistence. Limitations include unclear reactivation triggers. Challenges include F M c persistence decades postpartum. Future potential includes real-time imaging and barcoded lineage tracing.

Row 4: Clinical translation (H S C T and tolerance induction). Limitations include lack of standardized protocols. Challenges include N I M A-mismatched donors with an a G v H D risk H R of 0.1. Future potential includes N I M A-aware donor selection.

Row 5: Autoimmunity versus oncology. Limitations include conflicting evidence on protection versus lesions. Challenges include breast cancer patients having lower F M c frequency than controls. Future potential includes therapeutic exploitation of fetal cell immune surveillance.

Row 6: Maternal-neonatal interface. Limitations include incomplete cargo mapping of milk and placental E V s. Challenges include breastfeeding as a route of N I M A exposure. Future potential includes engineered E V s for tolerance induction.

Row 7: Ethical, regulatory and translational implications. Limitations include lack of regulatory frameworks. Challenges include F M c persistence without offspring consent. Future potential includes development of ethical frameworks and translational pipelines.

Navigate back to Table 4..
